# Foliar selenium application with compound auxiliaries enhances selenium accumulation, grain quality, and heavy metal detoxification in black wheat (*Triticum aestivum* L.)

**DOI:** 10.3389/fpls.2025.1652781

**Published:** 2025-11-10

**Authors:** Wenxia Pei, Mengya Dai, Ruoyan Yin, Jifeng Jiang, Chunming Chen, Xue Yin, Hongbao Wu, Jianfei Wang

**Affiliations:** 1 College of Resource and Environment, Anhui Science and Technology University, Fengyang, China; 2 Bengbu Bengshan District Agricultural Comprehensive Service Station, Bengbu, China

**Keywords:** selenium biofortification, black wheat (*Triticum aestivum*), selenomethionine, heavy metal detoxification, translocation efficiency, foliar fertilization

## Abstract

This study investigated the effects of foliar selenium (Se) application (either alone or in combination with compound adjuvants or microbial agents) on the yield, Se distribution, translocation, organic Se forms, and mineral composition in black wheat (*Triticum aestivum L.*). Using sodium selenite (Na_2_SeO_3_) as the Se source, five treatments were applied via unmanned aerial vehicle (UAV) spraying under optimal field conditions (30 L per 10 mu; clear, windless afternoons): CK (distilled water control), T1 (Na_2_SeO_3_ alone), T2 (Na_2_SeO_3_ + adjuvant), T3 (Na_2_SeO_3_ + microbial agent), and T4 (Na_2_SeO_3_ + adjuvant + microbial agent). Key findings revealed that T2 significantly increased the 1000-grain weight by 13.79% compared to CK, while other treatments showed no significant yield improvements. All Se treatments markedly elevated total Se content across plant tissues, with T1 and T2 achieving the highest and statistically similar Se accumulation in grains (grain Se content increased about 9–10 fold versus CK). T1 demonstrated the most efficient Se translocation from spikes to grains and husks. Organic Se speciation analysis identified selenomethionine (SeMet) as the predominant form in grains, with T1 yielding the highest SeMet concentration, while the addition of auxiliaries (T2, T3, T4) significantly reduced SeMet content compared to T1. Additionally, Se application enhanced essential mineral levels (Ca, Mg, Zn, Fe) while reducing toxic heavy metals (As, Cd, Pb). Notably, T2 was particularly effective in reducing Cd concentration to 0.032 mg/kg, meeting food safety thresholds. These results demonstrate a clear trade-off: foliar Se application alone (T1) optimizes nutritional quality via SeMet enrichment, whereas its combination with an adjuvant (T2) provides a balanced strategy, enhancing yield and Cd safety alongside robust Se biofortification. This integrated foliar application approach offers insights for balancing Se enrichment and heavy-metal mitigation in functional agriculture.

## 1 Introduction

Selenium (Se) is an essential redox-active micronutrient for living organisms that is found in both crystalline and amorphous forms in nature (Bisht et al., 2022; Titov et al., 2022). Although once regarded as toxic, subsequent research has highlighted its beneficial effects, prompting some scholars to refer to Se as the “miracle element of life” (Tan et al., 2016; Zafeiriou et al., 2022; Di et al., 2023). Se deficiency is linked to oxidative-related metabolic disorders, including cancer, cardiovascular diseases, diabetes, infertility, immune dysfunction, and cognitive decline (Guillin et al., 2019; Xie et al., 2021). Surveys across 13 provinces and cities in China indicate that the daily Se intake of urban and rural residents ranges from 26 to 32 μg, significantly below the recommended 50-200 μg/day (Yang et al., 2016; Roberto et al., 2018). Thus, the Se biofortification of staple foods is crucial for preventing diseases related to Se deficiency. The threshold for daily Se intake ranges between 40 and 400 μg/day, with intakes below 40 μg/day leading to malnutrition and intakes above 400 μg/day potentially causing toxicity (Gupta and Gupta, 2016). Therefore, scientifically balanced Se supplementation is essential. Because the human body cannot synthesize organic Se, dietary Se intake is vital for maintaining health. Se-enriched agricultural products, which primarily contain organic Se, are efficiently absorbed by the human digestive system. The Se biofortification of crops is a key strategy for increasing Se intake due to its high bioavailability and safety when consumed via agricultural products (Li et al., 2018; Zhou et al., 2018).

The Se biofortification of plants can be achieved through various methods, including the use of beneficial microorganisms (Ye et al., 2020; Yang et al., 2021), foliar applications of sodium selenite, sodium selenate, and potassium selenate (Duborska et al., 2022), and soil Se application (Wang et al., 2022a). Current biofortification efforts have successfully enhanced the Se content of a variety of crops, fruits, vegetables, and fungi, including rice (Zhang and Chu, 2022), soybeans (Silva et al., 2022), wheat (Liu et al., 2021; Lei et al., 2023), apples (Groth et al., 2020), Hericium erinaceus (Hu et al., 2020), Ganoderma lucidum (Xu et al., 2021), kale (Golob et al., 2020), and young leafy vegetables (Francini et al., 2023). The bioavailability and transfer of Se into the food chain are influenced by factors such as soil biophysical chemistry, competing ions, plant species/genotype, application method, and Se application rates (Fordyce, 2007; Lopes et al., 2017; Lessa et al., 2020). Notably, soils contaminated with heavy metals (e.g., Cd, Pb, and Ni) can affect Se mobility, plant uptake, and translocation, and conversely, Se has been reported to mitigate heavy metal toxicity in plants through antioxidative and chelation mechanisms (Chen et al., 2024; Zheng et al., 2024). Tropical soils are rich in Fe/Al oxyhydroxides and have a high capacity for Se retention (Lessa et al., 2016; Araujo et al., 2018), making foliar spray application an effective strategy to overcome Se sorption in soils and enhance the efficiency of biofortification (Zhu et al., 2017; Lessa et al., 2020).

Wheat is a staple crop for over one-third of the global population (Fernandes et al., 2018) and is recognized as the most efficient Se accumulator among common cereals (Poblaciones et al., 2014; Dinh et al., 2018). Wheat biofortification is thus a promising strategy to enhance dietary Se intake (Galinha et al., 2015). However, 63% of the wheat cultivated in China is deficient in Se, with an average concentration of 64.6 µg/kg, which is insufficient to meet daily human Se requirements (Liang et al., 2020). The agronomic Se biofortification of wheat is a viable approach to address this deficiency. Research indicates that Se application can significantly increase the Se content of wheat grain, with the effectiveness influenced by the application method, Se form, and application rate (Keskinen et al., 2010; Wang et al., 2019; Lara et al., 2019). Both selenate (Se^6+^) and selenite (Se^4+^) are widely used for plant biofortification, but they differ in uptake and metabolism. Selenate is highly mobile and efficiently translocated, whereas selenite is rapidly reduced and incorporated into organic Se compounds such as selenocysteine and selenomethionine, which are nutritionally valuable in cereal grains (White, 2016). Moreover, selenite has been shown to interact with heavy metals in soils, reducing their bioavailability and accumulation in plant tissues, thereby enhancing food safety (An et al., 2024). In this study, sodium selenite (Na_2_SeO_3_) was chosen as the selenium source for foliar application, Compared with selenate, selenite is less prone to runoff or volatilization, ensuring stable leaf absorption (Dinh et al., 2018). Furthermore, previous studies have confirmed the effectiveness of selenite in increasing selenium content in wheat under comparable conditions (Wang et al., 2022a; Di et al., 2023). However, existing studies predominantly focus on different forms of Se and application methods in common wheat, while limited research has focused on foliar spraying using Se compound auxiliaries.

Color-grained wheat genotypes, including blue, purple, and black varieties, differ from common red or white wheat in their anthocyanin content—antioxidant pigments present in the aleurone and pericarp. These colored genotypes often exhibit protein and amino acid levels that are at least 7% higher than those found in common wheat cultivars (Tian et al., 2018). Research by (Xia et al., 2020) demonstrated that purple-grained wheat could accumulate higher concentrations of Se, particularly organic Se, in its grain compared to common wheat. Additionally, color-grained wheat cultivars contain a variety of natural com-pounds that are beneficial to human health, offering the potential for producing value added, functional flour products with high nutritional value and antioxidant properties (Pasqualone et al., 2015).Considering their high antioxidant and chelating capacity, color-grained wheat may also provide enhanced protection against heavy metal stress, making them ideal candidates for studies combining Se biofortification and heavy metal detoxification.

While selenium (Se) biofortification via foliar application is widely recognized as an effective strategy to combat Se deficiency in cereal-based diets, the efficiency of foliar-applied Se is yet largely governed by its uptake, translocation, and assimilation within plants. This process can be significantly influenced by external auxiliaries, including adjuvants and microbial agents. Adjuvants can improve foliar Se absorption and internal translocation by reducing droplet surface tension, increasing adhesion to the leaf surface, and enhancing penetration through the cuticular barrier (Fernández and Eichert, 2009). In contrast, microbial agents, including beneficial rhizobacteria and endophytes, can form associative relationships with crop plants. These interactions serve to enhance the plants’ innate resistance against pathogens while also supporting their growth and developmental processes (Elnahal et al., 2022). Importantly, some microbial agents can also contribute to heavy metal immobilization in soil or plant tissues, further mitigating potential metal toxicity during Se biofortification (Wei et al., 2025).

This study was conducted under the hypothesis that integrating selenium (Se) with compound auxiliaries could enhance its foliar absorption and translocation by modulating both physicochemical and biological pathways of uptake. In this framework, surface-active adjuvants were expected to facilitate Se droplet adhesion, cuticular penetration, and retention on leaf surfaces through physicochemical enhancement, whereas microbial agents were anticipated to stimulate Se uptake and assimilation by improving nutrient metabolism and promoting beneficial plant–microbe interactions. To comprehensively evaluate these mechanisms, two categories of auxiliaries chemical (adjuvants) and biological (microbial agents) were selected as representative enhancers of Se utilization efficiency. Although their mechanisms differ, both aim to maximize Se bioavailability and translocation efficiency within the plant system. Accordingly, five treatments were established to assess their individual and synergistic effects on Se uptake, distribution, and biofortification: distilled water control (CK), Na_2_SeO_3_ alone (T1), Na_2_SeO_3_ + adjuvant (T2), Na_2_SeO_3_ + microbial agent (T3), and Na_2_SeO_3_ + both auxiliaries (T4). Furthermore, to elucidate the broader nutritional and environmental implications of Se biofortification, this study simultaneously examined the concentrations of essential mineral nutrients (Ca, Mg, Zn, Fe, Mn, Mo, Cu) and toxic heavy metals (As, Cd, Hg, Pb) in black wheat grains. This integrative approach aimed to determine the dual function of foliar Se application in enhancing grain nutritional quality while mitigating heavy metal accumulation under slightly contaminated yet agronomically representative soil conditions.

## 2 Materials and methods

### 2.1 Experimental design and plant growth conditions

The field experiment was conducted during the 2022–2023 cropping season in a wheat field at Qiyuan Street, Mengcheng County, Bozhou City, Anhui Province, China (33.304536°N, 116.549062°E). The region has a warm temperate semi-humid monsoon climate, with an average annual temperature of 14.8°C and rainfall of 900 mm. During the growing season, the mean temperature was 13.2°C (range: 2.8–25.6°C), and total precipitation was 580 mm, with 68% occurring from jointing to grain filling (March–May). Sunshine averaged 6.2 h day⁻¹, with maximum radiation during grain filling. No extreme weather events occurred during critical growth stages.

Soil samples (0–20 cm) were collected, air-dried, homogenized, and sieved (2 mm and 0.149 mm) for physical and chemical analyses following SD (2022). The soil properties were: pH 5.44, organic carbon 22.41 g/kg, alkali-hydrolyzable nitrogen 21.82 g/kg, available phosphorus 10.73 g/kg, available potassium 164.17 g/kg, total Se 0.51 mg/kg, and cadmium 0.64 mg/kg. Winter wheat (‘Liuzi Rye No. 1’) was sown in mid-to-late October 2022. Fertilizer rates were determined based on soil nutrient status and crop requirements to ensure adequate nutrient supply, included 750 kg/ha of compound fertilizer (15-15-15) and 150 kg/ha of urea (46.4% N), with 150 kg/ha ammonium nitrate phosphate (30-4-0) applied as topdressing.

A completely randomized design with three replicates per treatment was used. Each plot measured 10 mu (≈ 0.67 ha) and was managed uniformly. Five treatments were established: CK, foliar application of distilled water; T1, foliar application of sodium selenite; T2, foliar application of sodium selenite with an adjuvant; T3, foliar application of sodium selenite with a microbial agent; and T4, foliar application of sodium selenite with both an adjuvant and a microbial agent. During the rye grain-filling stage, sodium selenite (Na_2_SeO_3_) solution was applied as a foliar spray to assess selenium uptake and translocation. The spraying solution delivered a pure selenium dose of 15 g Se ha⁻¹, calculated from the selenium content in Na_2_SeO_3_ (45.6% Se by weight). Unmanned aerial vehicles (UAVs) evenly distributed the solution onto leaf surfaces via foliar spray after 3:00 PM under calm conditions, ensuring uniform coverage while minimizing drift or evaporation losses. In contrast, control plots received an equivalent volume of deionized water. The adjuvant is MOMENTIVE Silicone Activator, composed primarily of ethoxylated is octyl alcohol, ethoxylated C12–14 alkanol, and poly(alkyl) oxide silane. The microbial agent primarily consists of Tetragenococcus methyltrophus E26, Bacillus subtilis M173, and a blend of natural protein polypeptides. The adjuvant and microbial agent were added according to manufacturer instructions immediately before spraying. Control plots received an equal volume of deionized water. All fertilization, irrigation, and management practices were standardized across treatments to ensure comparability.

### 2.2 Sample collection

The harvested wheat (June 2023) underwent an initial cleaning process that included three washes with tap water to remove dust and impurities, followed by three rinses with deionized water, and then drying with absorbent paper. Each wheat plant was then divided into six parts: the roots, stems, leaves, spikes, glumes, and grain. The fresh weight (FW) of the roots was recorded before drying. Each replicate was placed in paper bags and dried at 105°C for 30 min, followed by drying at 70°C to a constant weight, after which the dry weight (DW) was obtained. All parts of the wheat plants were ground into powder and sieved through a 0.149-mm mesh to determine the total Se content.

### 2.3 Determination of Se content

For the determination of the Se content, wheat samples weighing 0.1000 g were passed through a 60-mesh sieve into a microwave digestion tank. Then, 7 mL of concentrated nitric acid and 2 mL of hydrogen peroxide were added to each sample and the samples were placed in a JUPITER-B microwave digestion instrument. After digestion, the digestion tank was transferred to a TK12 acid chaser and heated to 120°C. Upon reducing the liquid to approximately 1 mL, 5 mL of 6 mol/L hydrochloric acid was added. Once the digestion solution cleared, the volume was adjusted to 25 mL and the solution was left to stand. Then, 10 mL of the digestion solution was transferred to a 15-mL centrifuge tube, and 1 mL of 10% potassium ferricyanide solution and 2 mL of concentrated hydrochloric acid were added. The mixture was shaken thoroughly and the Se content was analyzed using a PF52 atomic fluorescence spectrophotometer.

The determination of organic Se and SeMet in the grains of black wheat is subject to experimental constraints and was therefore entrusted to the Geological Experimental Research Institute of Anhui Province for analysis.

### 2.4 Determination of trace elements

First, 0.1000 g of powdered wheat grain sample was accurately weighed using an analytical balance with a precision of 0.0001 g and placed in a microwave digestion vessel. Then, 6 mL of concentrated nitric acid and 2 mL of hydrogen peroxide were added, the lid of the digestion vessel was secured, and cold digestion was allowed to proceed overnight. The next day, the digestion vessel was placed on the fixed turntable of the microwave digestion system and heated for 1 h. After heating, the digestion vessel was removed and placed in an acid drive-off apparatus at 180°C. When the solution became clear and transparent, with approximately 1 mL remaining, it was removed and allowed to cool to room temperature. The solution was transferred to a 10-mL volumetric flask, the digestion vessel was rinsed three times with ultrapure water, and the residual solution was transferred to the volumetric flask as well. Ultrapure water was added to bring the solution up to a volume of 10 mL, after which the solution was mixed thoroughly and filtered through a disposable syringe filter (0.22-μm water soluble membrane) into a centrifuge tube for analysis. Inductively coupled plasma mass spectrometry (ICP-MS) was performed to determine the contents of trace nutrients (Ca, Mg, Zn, Mn, Mo, Cu, Fe) and heavy metals (As, Cd, Hg, Pb) in the processed wheat grain samples.

Different analytical techniques were employed for Se and other trace elements to ensure optimal detection accuracy, as atomic fluorescence spectrometry (AFS) offers higher sensitivity for Se, while ICP–MS allows simultaneous quantification of multiple elements.

### 2.5 Statistical analysis

Data processing was performed using Excel 2021, and variance analysis was conducted with SPSS 27.0 software (IBM, USA) using Duncan’s method for significance testing at p = 0.05. Histograms and Pearson’s correlation analysis were created using Origin 2024 software. The displayed data represent the mean values of three replicates.

The translocation factor (TF) was calculated using the following formula:


$$TF_{a/b}=TF_{a/b}=\frac{Ca}{Cb}$$

where TF_a/b_ represents the Se translocation factor from plant part b to part a, and C_a_ and C_b_ represent the Se content in part a and part b, respectively.

## 3 Results

### 3.1 Effects of Se application on the yield and its component factors

Se has been found to promote growth through improving the physiological characteristics of plants (Khan et al., 2023). Demonstrated that applying a certain concentration of Se can significant increased the yield of Black-Grained Wheat (Liu et al., 2021). In this study, foliar application of selenium-based compounds or microbial agents was shown to improve the 1000-grain weight of triticale to varying degrees. Notably, the 1000-grain weight under the T2 treatment reached 40.57 g, representing a significant increase of 13.79% compared to the control (CK). This treatment exhibited the most pronounced effect (**Table 1**). Although other treatments also resulted in increases in the 1000-grain weight of triticale, these improvements were not statistically significant. Additionally, all treatments led to some enhancement in rice yield, with increases ranging from 3.10% to 5.23%. Among these, the T2 treatment demonstrated the most substantial improvement; however, the increase was not statistically significant. Furthermore, none of the treatments significantly affected the number of panicles or the number of grains per panicle.

**Table 1:** The black wheat yield and its components under each treatment.

Treatment	Number of panicles (×10^4^/hm^2^)	Number of grains per panicle	1000 - grain weight (g)	Yield (kg/hm²)
CK	570.95 ± 25.73a	41.00 ± 2.65a	35.66 ± 2.36b	7077.64 ± 408.88a
T1	542.94 ± 24.12a	40.67 ± 3.06a	39.02 ± 2.58ab	7296.72 ± 232.39a
T2	536.27 ± 14.43a	40.33 ± 2.08a	40.57 ± 1.70a	7447.99 ± 177.97a
T3	561.61 ± 23.11a	42.00 ± 1.73a	36.99 ± 1.82ab	7408.33 ± 359.22a
T4	568.28 ± 10.59a	39.67 ± 3.06a	38.10 ± 3.14ab	7271.40 ± 299.03a

**Figure 1:**
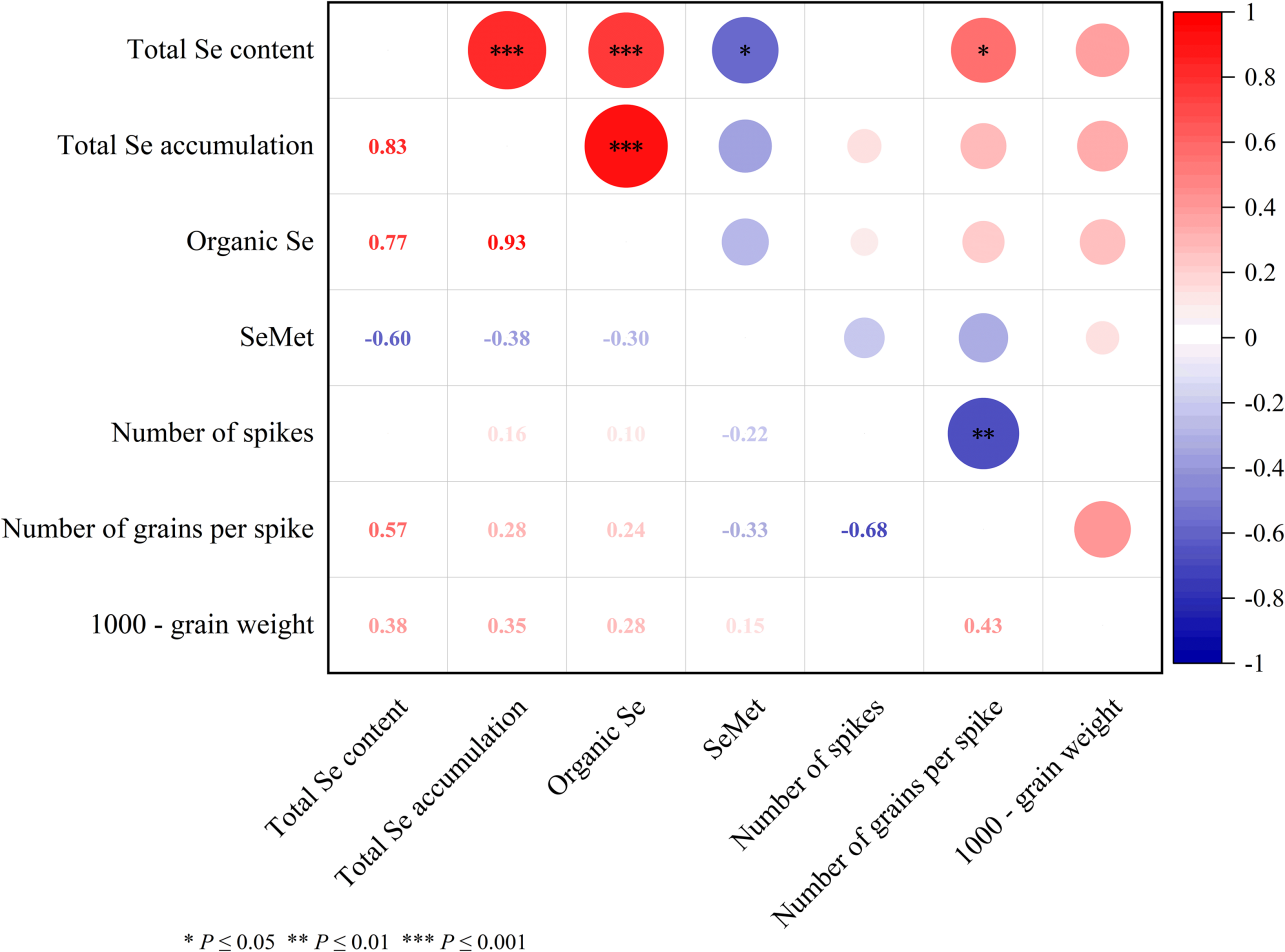
Correlation matrix between selenium species and yield-related traits in Mengcheng rye. Values represent Pearson correlation coefficients. Organic Se denotes organic selenium content; SeMet refers to selenomethionine. Significance levels are indicated as *p ≤ 0.05, **p ≤ 0.01, and ***p ≤ 0.001. Positive and negative coefficients indicate direct and inverse relationships, respectively. The matrix highlights the strong association between organic selenium forms and total selenium accumulation, as well as the divergent relationships between selenium species and yield components.

The correlation analysis between various selenium forms and yield-related traits in Mengcheng rye revealed significant relationships (**Figure 1**). Organic Se content showed a strong positive correlation with total Se accumulation (r = 0.93, p ≤ 0.001) and total Se content (r = 0.77, p ≤ 0.001). Similarly, a very high positive correlation was observed between total Se content and accumulation (r = 0.83, p ≤ 0.001). In contrast, SeMet exhibited moderate negative correlations with total selenium content and accumulation (r = -0.60, p ≤ 0.001). Among yield components, the number of grains per spike was positively correlated with total Se content (r = 0.57), while it showed a strong negative correlation with the number of spikes (r = -0.68). The 1000-grain weight was positively associated with all selenium metrics, though not always significantly. These results underscore the complex interplay between selenium speciation and agronomic performance in rye.

### 3.2 Total Se concentration and translocation factor of Se in wheat plants

**Figure 2:**
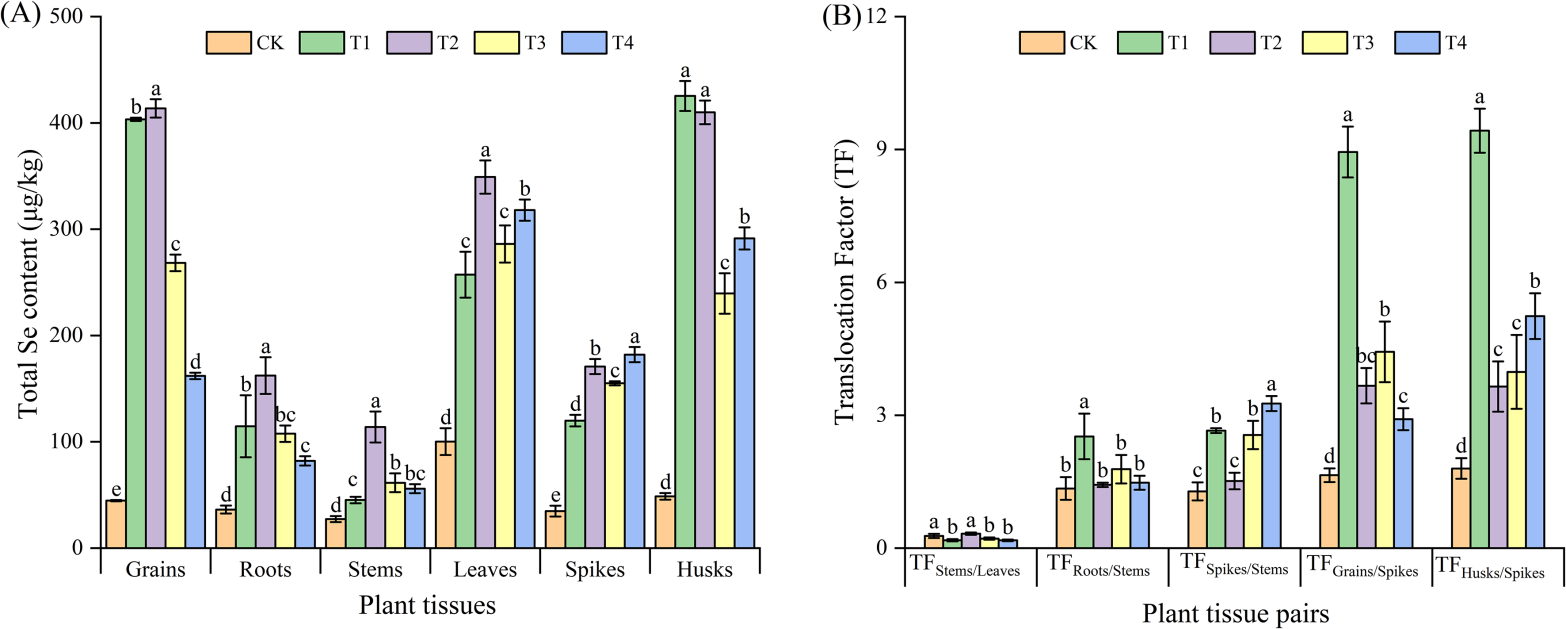
Total selenium (Se) content **(A)** and translocation factor (TF) **(B)** in different parts of triticale wheat. **(A)** Total Se content (µg/kg) in different plant parts under different treatments. Plant parts include seeds, roots, stems, leaves, spikes, and glumes. **(B)** TFs of Se between different plant parts under various treatments. CK, foliar application of distilled water; T1, foliar application of sodium selenite; T2, foliar application of sodium selenite with an adjuvant; T3, foliar application of sodium selenite with a microbial agent; and T4, foliar application of sodium selenite with both an adjuvant and a microbial agent. Bars represent the mean values, and error bars indicate the standard error (SE). Different lowercase letters above the bars indicate significant differences at P< 0.05.

The harvested wheat plants were divided into six parts, comprising the grain, roots, stems, leaves, spikes, and husks. It was observed that the foliar spraying of Se com-pound auxiliaries or microbial agents significantly (*P* < 0.05) increased the total Se con-tent in each part of the triticale wheat in comparison to CK (**Figure 2A**). The total Se contents in the grain, root, stem, and leaf tissues were the highest in the T2 treatment, while the total Se contents in the spike and husk tissues were the highest in the T4 and T1 treatments, respectively. The total Se contents in the grains under T1, T2, T3, and T4 treatments were 403.33 μg/kg (~9.03 times that of CK), 413.67 μg/kg (~9.26 times that of CK), 268.33 μg/kg (~6.01 times that of CK), and 162.00 μg/kg (~3.63 times that of CK), respectively.

The translocation factor (TF) can be used to reflect the translocation capacity of plants from source to sink (Dinh et al., 2019). (**Figure 2B**) shows the effects of different treatments on the TF of different parts of wheat. In general, comparison with the CK, the treatments remarkably enhanced the translocation of Se from the spikes to the grains and husks. Among these treatments, the most conspicuous effect was witnessed in the T1 treatment. For this particular treatment, the translocation factors reached 8.94 (TF_Grains/Spikes_) and 9.42 (TF_Husks/Spikes_). It is interesting to note that in the T1, T3, and T4 treatments, the values of TF_Spikes/Stems_ were significantly higher than those in the CK, while the values of TF_Stems/Leaves_ were significantly lower than those in the CK. In contrast, the T2 treatment demonstrated no significant difference from the CK in this regard. With respect to the TF_Roots/Stems_, the T1 treatment led to a significant increase in this value, which was approximately 1.87 times higher than that of the CK. However, no significant differences were observed for the T2-T4 treatments.

### 3.3 Total Se accumulation and accumulation distribution ratio in wheat plants

**Figure 3:**
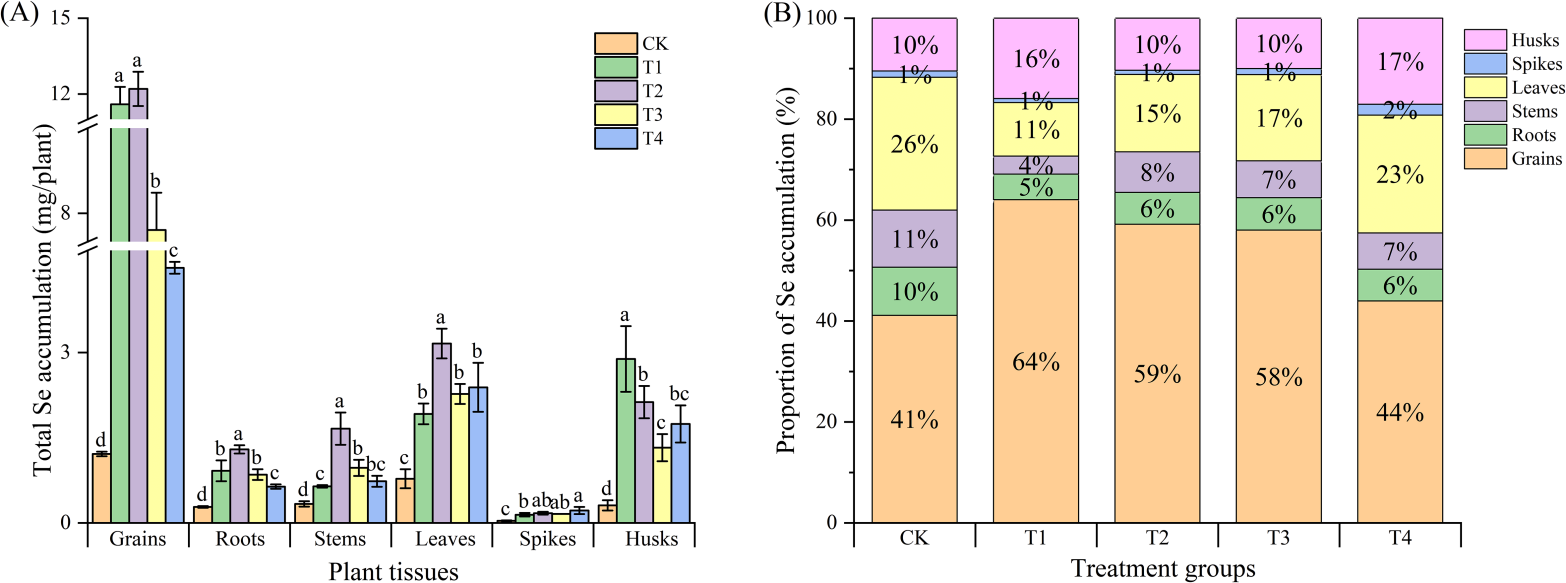
Total Se accumulation and distribution in different plant parts under various treatments. **(A)** Total Se accumulation (µg/plant) in different plant parts under different treatments. Plant parts include the seeds, roots, stems, leaves, spikes, and husks. Treatments are represented by distinct patterns. CK, foliar application of distilled water; T1, foliar application of sodium selenite; T2, foliar ap-plication of sodium selenite with an adjuvant; T3, foliar application of sodium selenite with a microbial agent; and T4, foliar application of sodium selenite with both an adjuvant and a microbial agent. Bars represent mean values, and error bars indicate the standard error (SE). Different lowercase letters above the bars denote statistically significant differences at P< 0.05. **(B)** Proportion of Se accumulation (%) in different plant parts under various treatments. The distribution is shown as a percentage of the total Se accumulation in seeds, roots, stems, leaves, spikes, and glumes for each treatment group. Each bar is segmented to display the relative contributions of each plant part to the total Se accumulation. Percentages within bars represent the proportion of Se in each plant part.

The trend of Se accumulation in various plant parts was consistent with the total Se content. The foliar spraying of Se compound auxiliaries or microbial agents significantly increased Se accumulation in all parts of black wheat. For the grains, roots, stems and leaves, the T2 treatment was the most effective, increasing Se accumulation by 10.04 times in grains, 4.61 times in roots, 4.97 times in stems, and 4.06 times in leaves com-pared to the control. While, the selenium accumulation in the spikes and husks was highest in the T4 and T1 treatments, with values of 0.22 μg (spikes) and 2.98 μg (husks), respectively (**Figure 3A**).

Further analysis of the proportions of Se accumulation in various wheat parts relative to the total Se accumulation in the whole plant revealed that, overall, foliar ap-plication with adjuvants or microbial agents increased the Se accumulation in black wheat grains to varying degrees (**Figure 3B**). The T1 treatment had the most significant effect, in-creasing Se accumulation by 23% compared to the control, followed by the T2 and T3 treatments, which increased Se accumulation by 18% and 17% compared to the control. However, compared to the control, all treatments reduced Se accumulation in the roots, stems, and leaves of black wheat to varying degrees, with the T1 treatment showing the most significant reduction. Interestingly, T4 treatment increased Se accumulation in the spikes and husks of black wheat, possibly because the addition of microbial agents inhibited Se translocation to the grains to some extent.

### 3.4 Effects of foliar spraying of Se on the organic Se contents in grains

**Figure 4:**
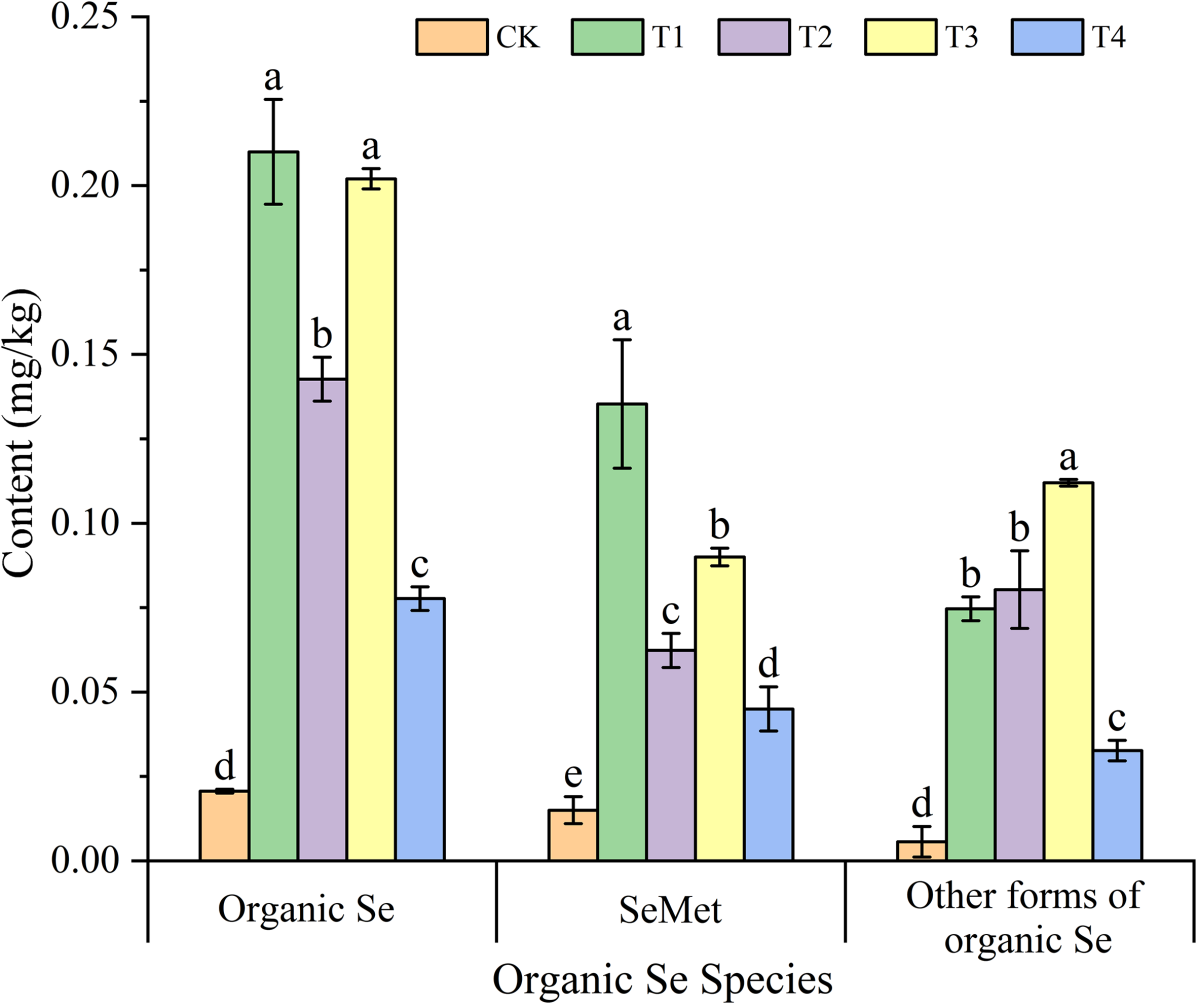
Effects of different treatments on the organic Se、SeMet and other forms of organic Se content of black wheat grains. The data represent the mean ± standard deviation (SD). Statistical significance among treatments is indicated by different lowercase letters (a, b, and c) within each column (P< 0.05).

**Figure 4** illustrates the impact of different treatments on the organic Se content in the grains of Mengcheng black wheat. Compared with the CK, foliar Se application significantly increased the organic Selenium, selenomethionine (SeMet), and other forms of organic selenium contents in the grains. Among the treatments, T1 treatment exhibited the highest organic Se content in the grains, reaching 0.21 mg/kg, which was 10.16 times that of the CK. Similarly, T3 demonstrated a comparable effect, with an organic Se content of 0.20 mg/kg, 9.77 times higher than the control. The organic Se con-tents in the T2 and T4 treatments were 6.9 times and 3.76 times that of the CK, respectively, which were significantly lower than those of T1 and T3. Furthermore, a significant difference was observed between T2 and T4. SeMet, as one of the main forms of organic Se in grains, displayed a trend consistent with the organic Se content. All treatments significantly enhanced the SeMet content in the grains. The T1 treatment produced the most notable increase, with a SeMet content of 0.14 mg/kg, 9.02 times that of the CK. T3 followed with a SeMet content of 0.09 mg/kg, which was significantly higher than those of the CK, T2, and T4 treatments, but lower than T1. Additionally, there was a significant difference in SeMet content between T3 and T4.

### 3.5 Effect of foliar Se spraying on the content of mineral elements and heavy metal contents in black wheat grains

The impacts of foliar Se spraying on the contents of various elements in black wheat grains are presented in **Tables 2**, **3**. **Table 1** summarizes the content of several mineral elements, including calcium (Ca), magnesium (Mg), zinc (Zn), manganese (Mn), molybdenum (Mo), copper (Cu), and iron (Fe), across different treatments. Compared with CK, the T1 treatment showed no significant effect on Ca, Mg and Fe contents and significant increases in Zn (18.60 mg/kg) and Mn (48.75 mg/kg) but reductions in Mo (0.07 mg/kg) and Cu (3.70 mg/kg). The T2 treatment exhibited the highest values of Ca (483.00 mg/kg), Mg (1287.50 mg/kg) and Fe (37.20 mg/kg), with significant increases in Ca, Mg, Zn, and Fe. The T3 and T4 treatments showed significant increases in Ca and Mg contents but reductions in Mo and Cu (**Table 2**).

**Table 2:** Mineral element contents of black wheat grains.

Treatment	Ca (mg/kg)	Mg (mg/kg)	Zn (mg/kg)	Mn (mg/kg)	Mo (mg/kg)	Cu (mg/kg)	Fe (mg/kg)
CK	394.50 ± 14.85c	1058.50 ± 36.06b	15.65 ± 0.35c	39.45 ± 1.91b	0.14 ± 0.01a	4.40 ± 0.10a	27.30 ± 3.99b
T1	413.50 ± 24.75bc	1163.00 ± 11.31ab	18.60 ± 0.00a	48.75 ± 3.04a	0.07 ± 0.00c	3.70 ± 0.00c	30.40 ± 1.60ab
T2	483.00 ± 35.36a	1287.50 ± 92.63a	18.05 ± 1.48ab	40.85 ± 5.16b	0.14 ± 0.00a	4.20 ± 0.20ab	37.20 ± 5.90a
T3	460.50 ± 7.78ab	1231.00 ± 14.14a	18.70 ± 0.28a	45.80 ± 1.27ab	0.10 ± 0.00b	3.90 ± 0.10bc	33.70 ± 0.10ab
T4	456.00 ± 1.41ab	1202.00 ± 36.77a	16.55 ± 0.21bc	38.35 ± 0.35b	0.11 ± 0.00b	3.90 ± 0.00c	33.20 ± 1.40ab

**Table 3:** Heavy metal contents of black wheat grain.

Treatment	As (µg/kg)	Cd (µg/kg)	Hg (µg/kg)	Pb (µg/kg)
CK	44.13 ± 4.24a	50.19 ± 0.81a	0.92 ± 0.21a	74.22 ± 8.49a
T1	27.25 ± 1.41b	48.66 ± 0.64a	0.71 ± 0.07a	24.17 ± 4.24b
T2	31.42 ± 3.54b	32.38 ± 1.41d	0.83 ± 0.05a	29.61 ± 5.90b
T3	31.33 ± 2.12b	42.25 ± 1.23b	0.74 ± 0.14a	27.85 ± 6.43b
T4	31.46 ± 2.12b	38.41 ± 0.71c	0.63 ± 0.00a	21.93 ± 1.41b

**Table 3** presents the concentrations of heavy metals, including arsenic (As), cadmium (Cd), mercury (Hg), and lead (Pb). Treatments with Se spraying generally resulted in decreased heavy metal concentrations. Compared to CK, T1 showed significant reductions in As, Cd, and Pb. The T2 treatment notably reduced the Cd content to 32.38 µg/kg. The T3 treatment exhibited slightly higher As content compared to the T2 treatment but lower As content than CK, and the T4 treatment had the lowest level of Pb (21.93 µg/kg), with reduced As and Cd contents (**Table 3**).

**Figure 5:**
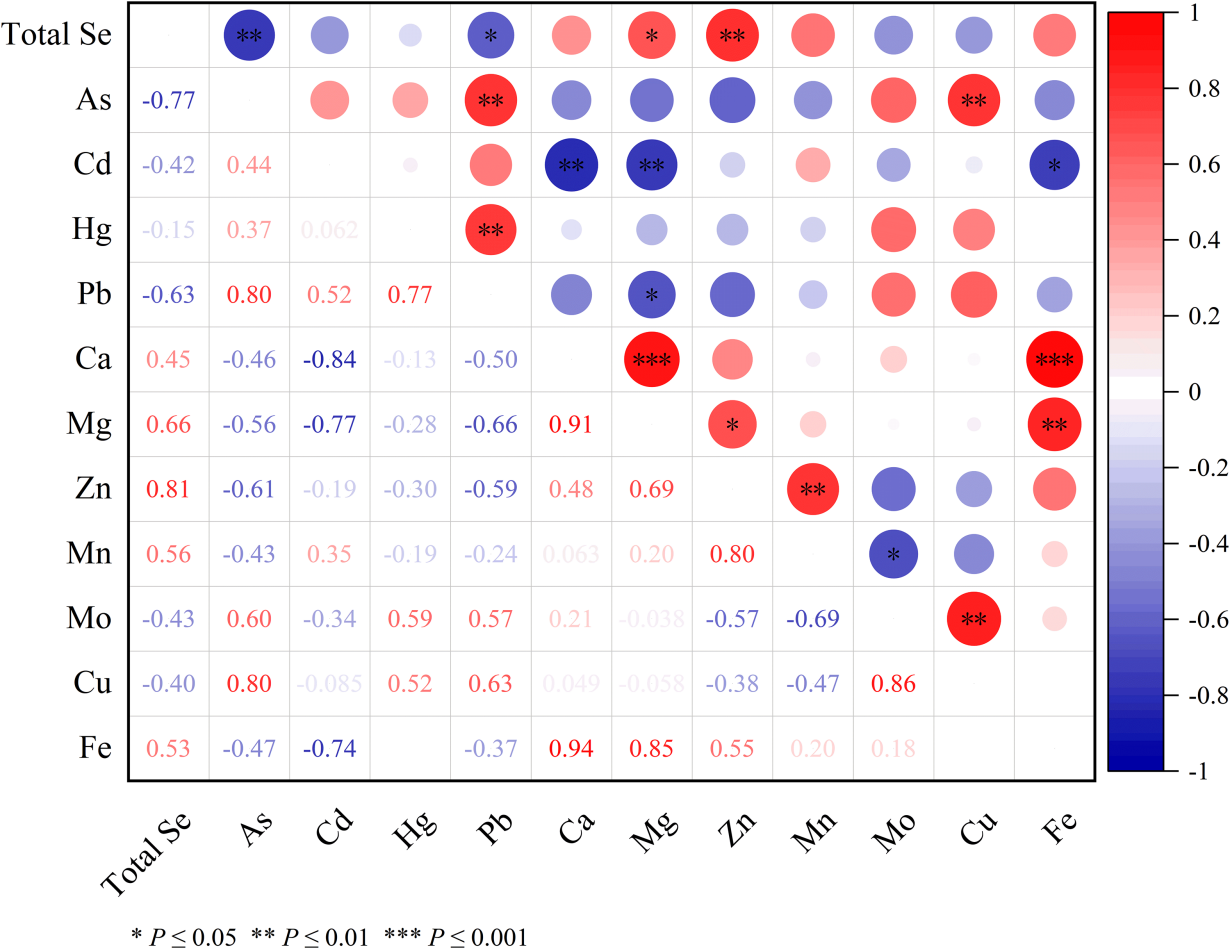
Correlation analysis among total Se content, mineral element and heavy metal contents. The values represent Pearson correlation coefficients. Positive correlations are shown in red, while negative correlations are shown in blue. The size and color intensity of the circles indicate the strength of the correlation. Asterisks denote the level of significance: *p ≤ 0.05, **p ≤ 0.01, ***p ≤ 0.001.

The correlation heatmap in **Figure 5** illustrates the relationships between total Se, mineral elements, and heavy metals in the grain samples. Total Se exhibited a strong positive correlation with Zn (r = 0.81, *p* ≤ 0.01) and Mg (r = 0.66, *p* ≤ 0.05), while exhibiting a significant negative correlation with As (r = -0.77, *p* ≤ 0.01) and Pd (r = -0.63, *p* ≤ 0.05). No significant relationship was found between total Se and other elements such as Cd, Hg, or Mn. Additionally, total Se exhibited significant negative correlations with As (r = -0.77, *p* ≤ 0.01) and Pb (r = -0.63, *p* ≤ 0.01), suggesting a potential antagonistic interaction between these elements. Among the mineral elements and heavy metals, As showed a robust positive correlation with Pb (r = 0.80, *p* ≤ 0.01) and Cu (r = 0.80, *p* ≤ 0.01), Hg also demonstrated a positive correlation with Pb (r = 0.77, *p* ≤ 0.001), while Ca showed positive correlations with both with Mg (r = 0.91, *p* ≤ 0.001) and Fe (r = 0.94, *p* ≤ 0.001). Mg was positively correlated with Zn (r = 0.69, *p* ≤ 0.05) and Fe (r = 0.85, *p* < 0.01), while Zn correlated with Mn (r = 0.80, p< 0.01) and Mo with Cu (r = 0.86, *p* < 0.05).In contrast, Cd showed significant negative correlations with Ca (r = -0.84, *p* ≤ 0.01), Mg (r = -0.77, *p* ≤ 0.01), and Fe (r = -0.74, *p* ≤ 0.05). Additionally, Pb was negatively correlated with Mg (r = -0.66, *p* ≤ 0.05), and Mn was negatively correlated with Mo (r = -0.69, p ≤ 0.05), indicating potential antagonistic interactions between these elements (**Figure 5**).

## 4 Discussion

The present study demonstrates that foliar Se application, particularly when combined with various adjuvants, has significant effects on both the mineral and heavy metal contents in black wheat grains, as well as on the overall plant growth and Se accumulation. A critical finding of this work is the distinct efficacy profile of sodium selenite applied alone (T1) compared to its application in combination with an adjuvant (T2) or microbial agent (T3/T4).

### 4.1 Effects on grain yield and components

Se has been well-documented for its growth-promoting properties, which are at-tributed to its improvement of plant physiological characteristic (Wang, 2024). Similar to the findings of (Liu et al., 2021) in tea, the findings of the present study suggest that appropriate Se treatments can enhance yield-related parameters in black wheat. In this study, the effects of foliar Se application on black wheat yield and its associated components were investigated (**Table 1**). The results demonstrate that foliar application of Se significantly increased the 1000-grain weight under the T2 treatment, with a 13.79% improvement compared to the CK, while the treatment resulted in notable increases in yield, these were not statistically significant. which was different to the previous results (Luo, 2019; Lara et al., 2019), which may suggest that other environmental factors, such as wheat type, soil organic matter and Se content, Se fertilizer source, processing period, and application method, could also play a role in modulating Se’s effect on yield (Yan et al., 2024).

The correlation analysis revealed a significant positive relationship between total Se content and 1000-grain weight (r = 0.57, *p* ≤ 0.05), suggesting that Se content in grains is likely linked to improved grain development. It is noteworthy that the significant increase in 1000-grain weight was unique to the T2 treatment. Conversely, the negative correlation between Se content and the number of spikes per unit area (r = -0.60, *p* ≤ 0.05) warrants further investigation, as it may indicate a trade-off between spike number and grain quality, a phenomenon that has been observed in dryland maize that higher yield, grain quality was concerned with Se fertilizer application (Wang et al., 2022b). Additionally, the strong negative correlation between the number of grains per spike and 1000-grain weight (r = -0.68, *p* ≤ 0.01) indicates a potential inverse relationship between grain size and number (**Figure 1**). This finding is consistent with earlier studies, However, more recent research has shown that in modern breeding varieties and selected introgression populations, where artificial selection plays a strong role, the relationship between the number of grains per spike and 1000-grain weight is either non-significant or positively correlated (Zhang, 2014).

### 4.2 Selenium accumulation and translocation in wheat plants

Foliar Se application notably increased Se content and accumulation in various plant tissues, this finding is in line with Soybean (Silva et al., 2023), wheat (Pasqualone et al., 2015), and rice (Lessa et al., 2020), similar studies demonstrating that foliar Se treatments can effectively enhance Se uptake and translocation within plants. Both T1 and T2 markedly elevated the Se content in wheat grains compared to the control (CK). However, no notable disparity emerged between T1 and T2 concerning grain Se concentration (**Figure 2A**), as their respective mean values (403.33 and 413.67 μg/kg) fell within the same statistical group, suggesting that the efficiency of foliar Se absorption is primarily governed by physicochemical Se speciation and plant genotype (Lara et al., 2019; Wang et al., 2022a), rather than surface adjuvants alone. Nevertheless, T2 demonstrated a notable advantage in enhancing yield parameters, particularly the 1000-grain weight, suggesting the adjuvant primarily contributed to yield improvement rather than additional Se enrichment. Overall, these findings demonstrate that both T1 and T2 treatments are remarkably effective for grain Se biofortification, whereas the T2 treatment displays significant yield-boosting potential, potentially offering complementary agricultural advantages.

The translocation factor (TF) analysis further highlighted the capacity of Se to move from source tissues (spikes) to sink tissues (grains and husks), particularly in the T1 treatment, which exhibited the highest TF values for both grains (TF_Grains/Spikes_ = 8.94) and husks (TF_Husks/Spikes_ = 9.42) (**Figure 2B**). This indicates that unassisted selenite is highly mobile post-absorption, efficiently reaching the edible portions. In contrast, the TF patterns for T2 were generally closer to the control, suggesting the adjuvant might favor initial leaf retention or a different internal redistribution pattern, without compromising the final grain Se load. This underscores that the adjuvant’s primary value regarding Se is not increasing the maximum grain Se concentration beyond that achievable by selenite alone, but potentially ensuring its more reliable deposition.

The Se accumulation in various wheat tissues was consistent with the observed Se content, with the T2 treatment leading to the highest increase in Se accumulation in grains, roots, stems, and leaves. This aligns with the findings of White (White, 2016). Interestingly, the T1 treatment showed the highest Se accumulation in spikes and husks, suggesting that the type of Se compound or microbial agent used may influence the preferential accumulation of Se in specific tissues. The reduction in Se accumulation in the roots, stems, and leaves, particularly in the T1 treatment, could be attributed to the increased translocation to the grains and spikes, as well as possible bioactive interactions between Se and other elements.

### 4.3 Organic Se content

Foliar Se treatments significantly enhanced the organic Se content in grains, particularly selenomethionine (SeMet), which is the primary form of organic Se in plants (**Figure 4**). Previous research also has shown that exogenous selenium can significantly increase the Organic Se content in oat grain (Li et al., 2021), and SeMet is the main form of organic Se in wheat grains (Li, 2017). The T1 treatment exhibited the highest increase in both total organic Se content (10.16 times that of the control) and SeMet (9.02 times the control). Furthermore, it is noteworthy that the T1 treatment (Se alone) was unequivocally superior for enriching grains with SeMet, yielding concentrations significantly higher than any treatment involving auxiliaries (T2, T3, T4) (**Figure 4**). This finding has major implications for nutritional quality. SeMet is the preferred form for human Se storage and is a key indicator of high-grade Se-biofortified food (White, 2016). The suppression of SeMet in auxiliary-treated plants suggests that these additives may alter the metabolic fate of absorbed selenite. They could potentially accelerate the reduction and assimilation of selenite in leaf tissues, leading to its incorporation into non-specific proteins or sequestration as other Se species, thereby reducing the pool available for remobilization as SeMet to the grain (Di et al., 2023). Therefore, for the primary goal of producing SeMet-enriched functional wheat, foliar application of sodium selenite without auxiliaries is recommended.

### 4.4 Impact on mineral elements and heavy metals

The effects of Se application on mineral elements and heavy metals in wheat grains were also evaluated. Overall, different treatments increased the content of calcium (Ca), magnesium (Mg), and iron (Fe) in the wheat grains to varying degrees (**Table 2**). The T2 treatment consistently showed the highest or among the highest values for these beneficial minerals, which aligns with earlier studies showing that Se supplementation can positively affect the uptake of essential nutrients in plants (He et al., 2004). However, the treatments also led to varying degrees of reduction in the concentrations of molybdenum (Mo), copper (Cu), and certain heavy metals, including arsenic (As), cadmium (Cd), and lead (Pb) (**Tables 2**, **3**). This suggests that Se may have a detoxifying effect on heavy metals in plants, potentially through competitive interactions at the root uptake sites or through the formation of less toxic Se-heavy metal complexes (Hussain et al., 2020). Regarding heavy metal mitigation, all Se treatments reduced As and Pb concentrations compared to CK. However, a crucial auxiliary-specific effect was observed for Cd, the T2 treatment exhibited the lowest cadmium (Cd) content (32.38 ± 1.41 µg/kg), representing a significant reduction of 33.46% and 35.49% compared with the T1 and CK treatments, respectively (**Table 3**). This indicates that while selenite itself has a general detoxification effect, the adjuvant provides an additional layer of protection specifically against Cd. The mechanism may involve the adjuvant promoting the formation of immobile Se-Cd complexes in the leaf apoplast or phloem, thereby hindering Cd’s translocation to the grain, a process that has been documented for Se in roots (Wang et al., 2023; Zheng et al., 2024). The differential effect on As and Pb could be attributed to their distinct chemical speciation and transport pathways in plants compared to Cd. The correlation heatmap further supports this, showing negative correlations between Se and heavy metals like As and Pb (**Figure 5**), which may indicate an antagonistic interaction, as observed in previous research (Gu et al., 2022).

## 5 Conclusion

In conclusion, this study provides a nuanced understanding of foliar Se biofortification in black wheat. The results delineate a clear trade-off between different application strategies. Foliar application of sodium selenite alone (T1) proved to be the optimal strategy for maximizing the nutritional quality parameter of SeMet content and for ensuring highly efficient Se translocation to the grain. In contrast, the combination of sodium selenite with an adjuvant (T2) presented a complementary profile, offering significant benefits in terms of 1000-grain weight (yield) and the most pronounced reduction in the toxic heavy metal Cd, while maintaining high total grain Se accumulation equivalent to T1. The use of the microbial agent showed intermediate effects. Therefore, the choice of Se application method should be goal-oriented: for producing high-SeMet functional food, Se alone is superior; for an integrated approach aiming to concurrently boost yield, achieve robust Se fortification, and enhance food safety (specifically against Cd), Se with an adjuvant is recommended. Future research should focus on optimizing Se application methods and concentrations to maximize these benefits while ensuring environmental safety elucidating the precise mechanisms by which adjuvants influence SeMet metabolism and Cd sequestration in plants.

## Data Availability

The original contributions presented in the study are included in the article/supplementary material, further inquiries can be directed to the corresponding author/s.
